# Antioxidant Enzyme Activities in Hepatic Tissue from Children with Chronic Cholestatic Liver Disease

**DOI:** 10.4103/1319-3767.61234

**Published:** 2010-04

**Authors:** Nagwa A. Ismail, Sawsan H. Okasha, A. Dhawan, Azza O. Abdel-Rahman, Olfat G. Shaker, Nehal A. Sadik

**Affiliations:** Department of Pediatrics, Medical Research Division, National Research Centre, Dokki; 1 Department of Pediatrics, Faculty of Medicine, Cairo University, Egypt; 2 Department of Pediatric Hepatology, King's College Hospital, London, UK; 3 Department of Medical Biochemistry, Faculty of Medicine, Cairo University, Egypt

**Keywords:** Catalase, cholestasis, glutathione peroxidase, oxidative stress, superoxide dismutase

## Abstract

**Background/Aim:**

To study the oxidative stress status in children with cholestatic chronic liver disease by determining activities of glutathione peroxidase (GPx), superoxide dismutase (SOD) and catalase (CAT) in liver tissue.

**Materials and Methods:**

A total of 34 children suffering from cholestatic chronic liver disease were studied. They were selected from the Hepatology Clinic, Cairo University, and compared with seven children who happened to have incidental normal liver biopsy. The patients were divided into three groups: extrahepatic biliary atresia (n=13), neonatal hepatitis (n=15) and paucity of intrahepatic bile ducts (n=6); GPx, SOD and CAT levels were measured in fresh liver tissue using ELISA.

**Results:**

In the cholestatic patients, a significant increase was found in mean levels of SOD, GPx and CAT in hepatic tissue compared to control children. The three enzymes significantly increased in the extrahepatic biliary atresia group, whereas in the groups of neonatal hepatitis and paucity of intrahepatic bile ducts, only GPx and CAT enzymes were significantly increased.

**Conclusion:**

Oxidative stress could play a role in the pathogenesis of cholestatic chronic liver diseases. These preliminary results are encouraging to conduct more extensive clinical studies using adjuvant antioxidant therapy.

Reactive oxygen species (ROS) are well recognized for playing a dual role as both deleterious and beneficial species. ROS are normally generated by tightly regulated enzymes, such as nitric oxide synthase. Beneficial effects of ROS (e.g., superoxide radical) occur at low/moderate concentrations and involve physiological roles in cellular responses to noxia, such as in defense against infectious agents, in the function of a number of cellular signaling pathways, and the induction of a mitogenic response. In contrast, overproduction of ROS [arising either from mitochondrial electron transport chain or excessive stimulation of NAD(P)H] results in oxidative stress.[1]

Oxidative stress is an abnormal phenomenon occurring inside our cells or tissues when production of oxygen radicals exceeds their antioxidant capacity. Excess of free radicals damage essential macromolecules of the cell, leading to abnormal gene expression, disturbance in receptor activity, proliferation or cell death, immunity perturbation, mutagenesis, protein or lipofuscin deposition.[2] Antioxidant enzymes catalyze decomposition of ROS. The three major antioxidant enzymes, superoxide dismutase (SOD), glutathione peroxidase (GPx) and catalase (CAT), differ from each other in structure, tissue distribution and cofactor requirement.[3]

The SOD is a group of metalloenzymes whose function appears to be protection of cells from the toxic effects of the endogenously generated superoxide radicals.[4] GPx is a selenocysteine-dependent enzyme that protects against oxidative injury. Endogenous activity of GPx is dependent on an adequate supply of the micronutrient selenium.[5] GPx enzymes are the most important hydrogen peroxide (H_2_O_2_)-removing enzymes in mammalian cells.[6] CAT is an essential enzyme in the decomposition of intracellular H_2_O_2_. It promotes the breakdown of H_2_O_2_ into water and oxygen without producing free radicals. CAT, which is located in peroxisomes, is found in essentially all aerobic cells.[4,5]

Disturbances in the antioxidant system could play a role in pathogenesis of chronic liver disease.[7,8] Liver disease in infancy has multiple etiologies. As reactive oxygen intermediates are involved in several types of tissue damage, it has been investigated whether different forms of liver disease in infancy are associated with increased free radical generation, using an indirect approach in which SOD (a free radical scavenger) activity is determined in the liver tissue.[9]

To our knowledge, no studies to evaluate the oxidative status in pediatric liver tissue have been performed so far; therefore, our aim was to study the oxidative status in children with cholestatic chronic liver disease using an indirect approach in which antioxidant enzymes, namely, GPx, SOD and CAT were determined in the liver tissue.

## MATERIALS AND METHODS

The present study included 34 children and adolescents (19 males and 15 females) suffering from cholestatic chronic liver disease. Patients were newly diagnosed and selected from Hepatology Clinic, New Children's Hospital, Cairo University, and were compared with a group of seven children who happened to have incidental “normal” liver biopsy (as a control group). Liver biopsy was indicated in these children as they exhibited persistent/intermittent elevations of liver enzymes for more than 6 months. Informed consent was obtained from the parents of children according to guidelines of the ethical committee of the National Research Centre, Dokki, Egypt.

The patients were divided into 3 groups:

Group I: Extrahepatic biliary atresia (EHBA) (n=13)

Group II: neonatal hepatitis (NH) (n=15)

Group III: paucity of intrahepatic bile ducts (PIBD) (n=6)

### Exclusion criteria

Patients with acute viral hepatitis, immune hepatitis, Wilson disease, concurrent conditions in which free radical production is elevated like other inflammatory processes that occur outside the liver or intake of antioxidant drugs affecting free radical scavenging such as vitamins C, A and E at the time of biopsy.

All cases were subjected to:

- Full history taking including personal history details, past and family histories; vaccination history
- Thorough clinical examination including anthropometric measurements including height and weight , vital signs, general and local abdominal examination
- Routine laboratory investigations including urine and stool analysis, complete blood count using Coulter counter, assessment of liver functions (total and direct bilirubin, alanine transaminase, aspartate transaminase and gamma glutamyl transpeptidase; serum total proteins and albumin; prothrombin time and concentration)
- Other laboratory investigations when needed, e.g., IGg
- Abdominal ultrasonography
- GPx, SOD and CAT levels in fresh liver tissue (cell-free homogenates) using Enzyme-Linked Immunosorbent Assay.

### Collection of samples

Liver tissue core was taken from each case using modified Minghini needle (sure cut). Each sample was divided into 3 portions, 1 for estimation of each enzyme and was stored at -80°C till assay.

### GPx assay

Liver biopsy was washed in phosphate buffer, pH 7.4. Then, the tissue was homogenized in 5 ml/g cold buffer, which is consisted of 50 mM Tris-HCl, pH 7.5, 5 mM EDTA, 1 nM dithiothreitol. The homogenate was centrifuged at 10,000*g* for 15 minutes at 4°C. The supernatant was removed for assay of GPx.

GPx activity was measured using Glutathione Peroxidase Assay Kit provided by Cayman Chemical Company, USA. GPx catalyzes the reduction of hydroperoxides, including H_2_O_2_, by reduced glutathione and functions to protect the cell from oxidative damage. With the exception of phospholipid-hydroperoxide GPx, a monomer, all GPx enzymes are tetramers of four identical subunits. Each subunit contains a selenocysteine in the active site, which participates directly in the two-electron reduction of the peroxide substrate. The enzyme uses glutathione as the ultimate electron donor to regenerate the reduced form of the selenocysteine.[10]

The Cayman Chemical Glutathione Peroxidase Assay Kit measures GPx activity indirectly by a coupled reaction with glutathione reductase (GR). Oxidized glutathione (GSSG), produced upon reduction of hydroperoxide by GPx, and is recycled to its reduced state by GR and NADPH.

Oxidation of NADPH to NADP+ is accompanied by a decrease in absorbance at 340 nm. Under conditions in which the GPx activity is rate limiting, the rate of decrease in the A340 is directly proportional to the GPx activity in the sample.[11]

### SOD assay

Liver biopsy was washed with 0.9% NaCl to remove red blood cells. The tissue was then blotted dry, weighted followed by homogenization in 200 µL buffer (0.05 M potassium phosphate and 0.1 mM EDTA, pH 7.8) and centrifuged at 15,000*g* for 30 minutes at 4°C.The supernatant was used for determination of SOD.

SOD was measured using Superoxide Dismutase assay kit provided by Oxis research, USA SOD-525 method. The BIOXYTECH is based on the SOD-mediated increase in the rate of autoxidation of 5,6,6a,11b-tetrahydro-3,9,10- trihydroxybenzo[c]fluorene R1 in aqueous alkaline solution to yield a chromophore with maximum absorbance at 525 nm.[12]

Interference due to mercaptans (RSH) such as reduced glutathione, is controlled by pretreating samples with 1-methyl-2-vinylpyridinium R2, which directly eliminates mercaptans by means of a fast alkylation reaction. The kinetic measurement of the 525 nm absorbance change is performed after the addition of R1.

The SOD activity is determined from the ratio of the autoxidation rates in the presence (Vs) and in the absence (Vc) of SOD. The Vs/Vc ratio as a function of SOD activity is independent of the type of SOD (Cu/Zn-SOD, Mn-SOD, Fe-SOD) being measured.[12] One SOD-525 activity unit is defined as the activity that doubles the autoxidation rate of the control blank (Vs/Vc=2).

### CAT assay

Biopsy was washed with phosphate buffer, pH 7.4, to remove red blood cells. The tissue was then blotted dry, weighted followed by homogenization in 1.5 ml cold buffer (50 mM potassium phosphate and 1 mM EDTA, pH 7) and centrifugation at 10,000*g* for 15 minutes at 4°C was done. The supernatant was used for the assay.

Catalase assay kit provided by Cayman Chemical Company, USA. CAT is a ubiquitous antioxidant enzyme that is present in most aerobic cells. CAT is involved in the detoxification of H_2_O_2_. This enzyme catalyzes the conversion of two molecules of H_2_O_2_ to molecular oxygen and two molecules of water (catalytic activity). CAT also demonstrates peroxidatic activity, in which low-molecular-weight alcohols can serve as electron donors, while the aliphatic alcohols serve as specific substrates. In human beings, the highest levels of CAT are found in the liver, kidney and erythrocytes, where it is believed to account for the majority of H_2_O_2_ decomposition.

The Cayman Chemical Catalase Assay Kit utilizes the peroxidatic function of CAT for determination of enzyme activity. The method is based on the reaction of the enzyme with methanol, in the presence of an optimal concentration of H_2_O_2_ .The formaldehyde produced is measured spectrophotometrically with 4-amino-3-hydrazino-5- mercapto-1,2,4-trizazole as the chromogen. The assay can be used to measure CAT activity in plasma, serum, erythrocyte lysates, tissue homogenates and cell lysates.[13]

### Statistical analysis

SPSS for Windows, version 7.0, computer program was used for statistical analysis. *P*<0.05 was considered statistically significant. One-way analysis of variance followed by post hoc comparison procedures were used to compare between three or more independent means. The *t* test was used to compare between two independent means. Pearson correlation coefficient (*r*) was used to measure the linear relationship between two quantitative variables. Data are represented as the mean±standard deviation.

## RESULTS

Descriptive data of the children included in the study are represented in Table 1. The mean levels of SOD, GPx and CAT enzymes in hepatic tissue of cholestatic patients and control children are shown in Table 2. A significant increase in mean levels of SOD, GPx and CAT was found in hepatic tissue of cholestatic patients compared with that in control children. In Table 3 the mean levels of the three enzymes in the three cholestatic patient groups are shown and compared to control children. The three enzymes were significantly increased in the extrahepatic biliary atresia group, whereas in the groups of neonatal hepatitis and paucity of intrahepatic bile ducts, only GPX and CAT enzymes were significantly increased. Studying the different correlations between liver function tests (total bilirubin, direct bilirubin, alanine transaminases, aspartate transaminases, albumin, prothrombin time, prothrombin concentration, alkaline phosphatase and gamma glutamyl transferase) and the three enzymes, SOD, GPX and CAT, in cholestatic patients revealed the presence of a positive significant correlation only between ALT and SOD levels (*r*=0.419, *P*≤0.05).

**Table 1:** Descriptive data of studied children

**Variable**	**Cholestasis group (n=34)**	**Control children (n=7)**
Age (yr) (mean±S.D.)	0.53±1.21 (range: 0.08-7)	3.62±3.17 (range: 0.66-7)
Sex (male/female)	19/15	3/4
Physical signs (number/%)		
Jaundice	34 (100)	0 (0)
Edema of lower limbs	0 (0)	0 (0)
Clubbing of fingers	1 (2.9)	0 (0)
Local abdominal signs:		
Diverication of recti	4 (11.8)	0 (0)
Hepatomegaly	7 (20.6)	0 (0)
Splenomegaly	4 (11.8)	0 (0)
Ascites	1 (2.9)	0 (0)
Hematological findings (mean±SD)		
Hb (gm/dl)	9.61±1.63	10.74±1.26
Platelets (×1000/mm^3^)	355.9±126.9	387.1±77.3
WBCs (×1000/mm^3^)	10.82±4.24	7.59±2.51
Liver function tests (mean±SD)		
Total bilirubin (mg/dl)	11.49±8.13*	1.04±1.2
Direct bilirubin (mg/dl)	5.73±4.57*	0.4±0.5
ALT (U/L)	162.7±153.5	36.2±20.8
AST (U/L)	231.5±158.6	59.1±32.7
GGT (U/L)	342.9±444.5	46.2±51.7
Albumin (g%)	3.57±0.75	4.55±0.5
PT (sec)	12.36±1.09	12.2±0.9
PC (%)	92.8±11.4	94.8±7.7

* = Significant difference (*P*<0.05) versus controls

**Table 2:** Comparison between mean levels (±SD) of superoxide dismutase, glutathione peroxidase and catalase (CAT) enzymes in children with cholestatic chronic liver disease and control children

**Variable**	**SOD (U/mg Ptn)**	**GPx (nmol/min/ml)**	**CAT (nmol/min/ml)**
Cholestasis group (n=34)	0.83±0.45*	66.77±12.31*	9.73±1.46*
Control children (n=7)	0.33±0.15	10.62±6.68	7.24±1.74

* = Significant difference (*P*<0.05) between patient group and control children, SOD= Superoxide dismutase; GPx= Glutathione peroxidase

**Table 3:** Comparison between mean levels (±SD) of superoxide dismutase, glutathione peroxidase and catalase (CAT) enzymes in different cholestasis groups and control children

**Variable**	**SOD (U/mg Ptn)**	**GPx (nmol/min/ml)**	**CAT (nmol/min/ml)**
EHBA (n=13)	0.97±0.52*	70.06±12.27*	10.11±1.74*
NH (n=15)	0.70±0.40	62.02±12.35*	9.26±1.48*
PIBD (n=6)	0.84±0.36	72.48±8.10*	10.00±1.58*
Control Children (n=7)	0.33±0.15	10.62±6.68	7.24±1.74

* = Significant difference (*P*<0.05) between patient group and control children, SOD= Superoxide dismutase; GPx= Glutathione peroxidase; EHBA= Extrahepatic biliary atresia; NH= Neonatal hepatitis; PIBD= Paucity of intrahepatic bile ducts

## DISCUSSION

Oxidative stress is a major pathogenetic event occurring in several liver disorders ranging from metabolic to proliferate ones, and is a major cause of liver damage in ischemia/ reperfusion during liver transplantation.[14] Many studies have shown that oxidative stress takes part in the pathogenesis of cholestasis by way of cytokines[15–18] and lipid peroxidation is responsible for the tissue injury in cholestasis.[19]

Our study showed a significant increase in hepatic tissue SOD, GPx and CAT activities in patients with cholestasis compared with control group [Table 2]; SOD, GPx and CAT were significantly increased in the EHBA group, whereas GPx and CAT were significantly increased in both NH and PIBD groups [Table 3]. Many studies in human beings and rats have been conducted to clarify the relationship between oxidative stress and antioxidants in the liver. In human beings, when suffering from free oxygen radicals, a complex defense system is activated. This system includes GPx, SOD, CAT, glutathione, glutathione reductase and vitamins.[20] Impairment of the bile flow is likely to result in the accumulation of toxic hydrophobic bile salts within the hepatocytes, with consequent injury caused by their detergent effect. Furthermore, bile salts can cause mitochondrial dysfunction by interfering with electron transport with consequent H_2_O_2_ and superoxide formation.[21]

In agreement with our results, Ercin *et al*[20] found increase in the levels of erythrocyte GPx and CuZnSOD in adult patients with cholestasis versus controls; however, their results were statistically nonsignificant. Broide *et al*[9] showed a significant increase in SOD levels in the liver of extrahepatic biliary atresia and Alagille patients, whereas nonsignificant increase was noted among neonatal hepatitis patients. They explained that SOD, a key enzyme in free radical protection, increases significantly in the liver tissue of infants with cholestatic liver disease due to bile duct damage, suggesting that products of free radical reactions are involved in the pathogenesis of these disorders. A significant increase in serum SOD activity has also been reported in another study in adult patients.[22]

Contrary to our results, Togashi *et al*[23] studied the levels of ZnSOD, CuSOD and CAT in liver and found them to be low and it was suggested that this was related to the effect of oxidative stress. In another recent study, a marked decrease in the antioxidant status was observed in serum and neutrophil homogenate of patients with chronic liver diseases in comparison with healthy subjects.[24] This study concluded that deficient antioxidant defense mechanisms may lead to excess oxygen free radical formation that promotes pathological processes in the liver.

In conclusion, the current study demonstrates that GPx, CAT and SOD levels are increased in hepatocytes of patients with chronic cholestasis. The significant increase in their levels may point to their role as key enzymes in the protection of the liver from the hazardous products of free radical reactions, and may reflect an appropriate activity of antioxidant barrier enzymes as a response to increased oxidative stress. Better knowledge of the redox regulation may have important clinical ramifications in understanding the pathogenesis of liver diseases and developing therapeutic approaches. These preliminary results are encouraging to conduct more extensive clinical studies combining antioxidant therapy with various treatments of chronic liver diseases.
